# Aqueous Flare Changes in Ex-PRESS Glaucoma Shunt Eyes After 4.7 Tesla High-Field Magnetic Resonance Imaging

**DOI:** 10.1167/tvst.12.5.3

**Published:** 2023-05-01

**Authors:** Ayaka Edo, Momoko Nakamura-Shibasaki, Takayuki Tamura, Kazuyuki Hirooka, Yoshiaki Kiuchi

**Affiliations:** 1 Department of Ophthalmology and Visual Sciences, Graduate School of Biomedical Science, Hiroshima University, 1-2-3 Kasumi, Minami-Ku, Hiroshima, Japan; 2 Department of Radiology, Hiroshima University Hospital, 1-2-3 Kasumi, Minami-Ku, Hiroshima, Japan

**Keywords:** flare count, Ex-PRESS, glaucoma, high-field MRI

## Abstract

**Purpose:**

Ex-PRESS glaucoma shunt stainless steel devices have been used worldwide for glaucoma treatment. The purpose of this study was to evaluate the safety of high-field magnetic resonance imaging (MRI) for Ex-PRESS–inserted eyes.

**Methods:**

Using rabbits, we performed Ex-PRESS shunt surgery in one eye in each rabbit and divided the rabbits into MRI and non-MRI groups. In the MRI group, 1 week after Ex-PRESS shunt surgery under low specific absorption rate (SAR) conditions and 1 week later under high SAR conditions, high-field 4.7-Tesla MRI was performed. Aqueous flare counts were measured before and after the Ex-PRESS shunt surgery and each MRI examination. The rabbits in the non-MRI group received only general anesthesia, and aqueous flare counts were measured as for those of the MRI group. Aqueous flare counts were expressed in photon counts per millisecond.

**Results:**

No dislocation of the Ex-PRESS shunt device was observed after MRI. The flare count ratio (MRI/non-MRI) in Ex-PRESS–inserted eyes 2 hours after high SAR MRI increased significantly compared with that before MRI (0.8 ± 0.3 vs 2.7 ± 0.8; pre-high SAR MRI vs 2 hours after high SAR MRI, respectively; *P* = 0.01). The day after MRI, the flare count improved spontaneously to the same level as that before MRI.

**Conclusions:**

Our results indicate that high-field MRI can be performed relatively safely after Ex-PRESS shunt surgery.

**Translational Relevance:**

This study demonstrates the safety of high-field MRI for Ex-PRESS–inserted eyes using a rabbit model.

## Introduction

Glaucoma is the leading cause of irreversible blindness in the world, with a prevalence of approximately 3.5% of people between the ages of 40 and 80 years, worldwide.^1^ Glaucoma is an intraocular pressure (IOP)-dependent disease, and the only established treatment is to lower the IOP.^2^ Topical medication, laser therapy, and surgery have been performed to lower IOP, depending on the patient's condition.^3^ Trabeculectomy is the gold standard, the most widely used approach in glaucoma surgery,^4^ and its IOP-lowering effect is well-established.^5^^,^^6^ Ex-PRESS (Alcon, Fort Worth, TX) minishunt surgeries have been performed worldwide.^7^ The principle of IOP reduction is the same as that in trabeculectomy. However, in Ex-PRESS shunt surgery, instead of resection of the scleral block and peripheral iridectomy, a short implant made of stainless steel is inserted under the scleral valve into the anterior chamber.^8^ Because Ex-PRESS insertion stabilizes aqueous humor outflow compared with traditional trabeculectomy, the reported frequency of postoperative hypotony is lower with Ex-PRESS shunts than with trabeculectomy.^9^ Additionally, there is no need for procedures such as iridectomy and scleral block resection with Ex-PRESS implantation, which has been shown to avoid intraoperative bleeding and potentially decrease postoperative inflammation.^10^ Indeed, some meta-analyses based on clinical data have shown that the Ex-PRESS shunt can decrease the frequency of postoperative interventions and anterior chamber bleeding while providing good IOP control that is comparable with that with trabeculectomy.^11^^,^^12^

The Ex-PRESS device is composed of 316L stainless steel, which is resistant to corrosion and impervious to magnetic fields.^13^ During magnetic resonance imaging (MRI) of patients with metal implants, there is a risk of migration of the implanted metal and burns on the metal. This risk increases as the magnetic field is increased during MRI.^14^ The safety of 1.5- and 3-Tesla (T) MRI in Ex-PRESS–inserted eyes has been demonstrated. It has been shown that Ex-PRESS shunts implanted in human cadaveric eyes did not move in 1.5- or 3-T magnetic fields.^15^ Clinical cases with Ex-PRESS implants have also shown that Ex-PRESS dislocation did not occur after imaging with 1.5- or 3-T MRI systems.^13^^,^^16^ It has also been shown that the maximum temperature increase was only 0.3°C when the Ex-PRESS was scanned by 1.5- or 3-T MRI under standard cerebral imaging conditions.^17^

Recently, the use of high-field MRI (>3 T) has become increasingly widespread, which enables more detailed organ delineation and more accurate diagnostic imaging compared with low-field MRI.^18^ It has been demonstrated that the Ex-PRESS was fully displaced to the limits of the petri dish when the Ex-PRESS was placed in a 4.7-T high-field MRI machine.^17^ Temperature increases and microscopic Ex-PRESS movements can cause intraocular inflammation. Ex-PRESS shunt surgery is widespread worldwide; therefore, it is important to evaluate the safety of high-field MRI for patients with Ex-PRESS–inserted eyes. In the present study, we focused on intraocular inflammation in the anterior chamber after Ex-PRESS implantation and MRI. To test the safety of high-field MRI in Ex-PRESS–inserted eyes, we performed 4.7-T high-field MRI of Ex-PRESS–inserted rabbit eyes and compared the degree of aqueous flare before and after the high-field MRI.

## Methods

### Animals

Six pigmented male rabbits (Dutch breed; weighing 2.0–2.5 kg; Hiroshimajikken, Hiroshima, Japan) were used for the experiments. Ex-PRESS shunt surgery was performed on only one eye of each rabbit. The rabbits were divided into two groups (*n* = 3 in each group) comprising an MRI group (eyes with MRI examination) and a no-MRI group (eyes without MRI examination). The same observations and examinations were performed for both eyes in the rabbits in each group. The animals were housed and treated in accordance with the ARVO Statement for the Use of Animals in Ophthalmic and Vision Research. All procedures were performed in accordance with the guidelines of the Committee on Animal Experimentation and the Committee of Research Facilities for Laboratory Animal Science, as well as the Natural Science Center for Basic Research and Development of Hiroshima University. All animals in this study were housed for at least 7 days before the experiment in an animal housing facility with a room temperature of 23°C and a 12-hour light/dark cycle (lights on at 8:00 am).

### Ex-PRESS Shunt Surgery

All surgical procedures were performed under sterile conditions. Ex-PRESS shunt devices were provided by the manufacturer (Alcon Japan, Tokyo, Japan). The rabbits were sedated with an intramuscular injection of 40 mg/kg medetomidine and 1 mg/kg butorphanol. Anesthesia was maintained after intravenous catheterization with an intravenous injection of 50 mg/kg pentobarbital. The right eye was cleansed with topical 0.5% povidone-iodine, and a lid speculum was placed to hold the lids open. A 7-mm conjunctival incision was made along the superior corneal limbus with spring microscissors to make a fornix-based conjunctival flap. A 25G needle was inserted 1.2 mm from the corneal limbus into the anterior chamber, underneath the conjunctival flap. The Ex-PRESS shunt device was then placed in the anterior chamber through the ostium created by the needle. After fixation of the Ex-PRESS device, the conjunctival peritomy was closed using 10-0 nylon with wing sutures. Ofloxacin eye ointment (Tarivid; Santen, Osaka, Japan) was applied at the end of the surgery, and no additional medications were administered postoperatively. The procedure required approximately 1 hour, including induction of anesthesia.

### MRI Examination

All experiments were performed with a 4.7-T superconducting magnet system (BioSpec47/40USR; Bruker BioSpin, Ettlingen, Germany) with a transmit/receive orthogonal volume coil (154 mm inner diameter). The rabbits underwent rapid acquisition of refocused echo imaging (repetition time, 4000 ms; echo time, 80 ms; field of view, 10 × 10 cm; image matrix, 256 × 256; slice thickness, 3 mm; and number of slices, 20) under the two conditions shown in Table 1. Two types of models were prepared: a low specific absorption rate (SAR) model with rapid acquisition of refocused echo factor of 3 and 1 average acquisition, and a high SAR model with a rapid acquisition of refocused echo factor of 15 and 14 average acquisitions. Before the animal experiment, we irrigated each sequence with 500 mL of saline and measured the temperature. The SARs were calculated on the basis of heat change in accordance with the report on the calculation of specific heat in seawater.^19^ The calculated SARsof the low SAR model and the high SAR model were 2.7 W and 20.0 W, respectively (Table 1).

**Table 1.** MRI Scanning Conditions

	Low SAR	High SAR
SAR (W/kg)	2.7	20
RARE factor	3	15
Average acquisition	1	14
Scan time (min)	5	15

MRI, magnetic resonance imaging; RARE, rapid acquisition of refocused echo.

Radiofrequency exposure produces an electric current in the body, creating a thermal effect owing to the absorption of electromagnetic wave energy. SAR is a measure of this thermal effect, usually indicated in watts per kilogram.^20^ International standards and safety guidelines recommend that SAR values not exceed 3.2 W/kg during head MRI because higher SARs are associated with increased heat generation and more frequent biological effects.^14^ In this study, the SAR value in the low SAR model was 2.7 W/kg, which is within the recommended limit, whereas in the high SAR model, the SAR value was very high at 20 W/kg.

### Anesthesia for MRI

Before the MRI examination, the rabbits were sedated with an intramuscular injection of 40 µg/kg medetomidine and 1 mg/kg butorphanol. Five minutes after sedation, the rabbits were masked and immobilized with isoflurane at 100 mL/min for 3 minutes in the surgical preparation room. The rabbits were then moved to the MRI room, and anesthesia was maintained with inhalational isoflurane at 60 mL/min. Inhalational anesthesia was maintained for 30 minutes for imaging under low SAR conditions and for 80 minutes under high SAR conditions. In the non-MRI group, anesthesia was administered at the same doses and concentrations as those in the MRI group and MRI was not performed.

### Aqueous Flare Measurement

Aqueous flare was quantified by photometric measurements using a laser flare meter (FM600; Kowa, Tokyo, Japan). Aqueous flare counts were expressed in photon counts per millisecond. The rabbit eye was placed in the center of the measurement section with no mydriasis and manually suppressed. Measurements were taken in an area measuring 0.3 × 0.5 mm at approximately the center of the anterior chamber. Measurements were taken by the same examiner; five measurements were taken, and the average value was used in the analysis.

Preoperatively, aqueous flare was measured at 11:00 am, and the Ex-PRESS implant surgery was performed at noon. Aqueous flare was measured 2 hours after surgery and at 1:00 pm on postoperative days 1 and 2. One week after the surgery, after flare measurement at 1:00 pm, MRI was performed under low SAR conditions. Aqueous flare was measured 2 hours after a low-SAR MRI and at 1:00 pm on days 1 and 2 after a low-SAR MRI. One week after low-SAR MRI, after flare measurement at 1:00 pm, MRI was performed under high-SAR conditions. Aqueous flare was measured 2 hours after high-SAR MRI and at 1:00 pm on days 1 and 2 after high-SAR MRI. In the non-MRI group, flare measurements were taken at the same times as those for the MRI group, but without performing MRI.

### Statistical Analyses

The data were expressed as mean ± standard deviation. Non–Ex-PRESS-inserted eyes acted as controls. To analyze the effect of MRI scanning on aqueous flare counts, we compared the MRI group/non-MRI group ratios for the aqueous flare counts in each of the Ex-PRESS and non–Ex-PRESS-inserted eyes. The time course changes in aqueous flare were compared by Dunnett's test using pre-MRI values as a reference. The aqueous flare count ratios between the Ex-PRESS and non–Ex-PRESS-inserted eyes at each time point were compared by the Student *t*-test. All statistical analyses were performed using JMP pro 16.0.0 (SAS Institute Inc., Cary, NC), and a *P* value of less than 0.05 was considered statistically significant.

## Results

**Figure 1.**
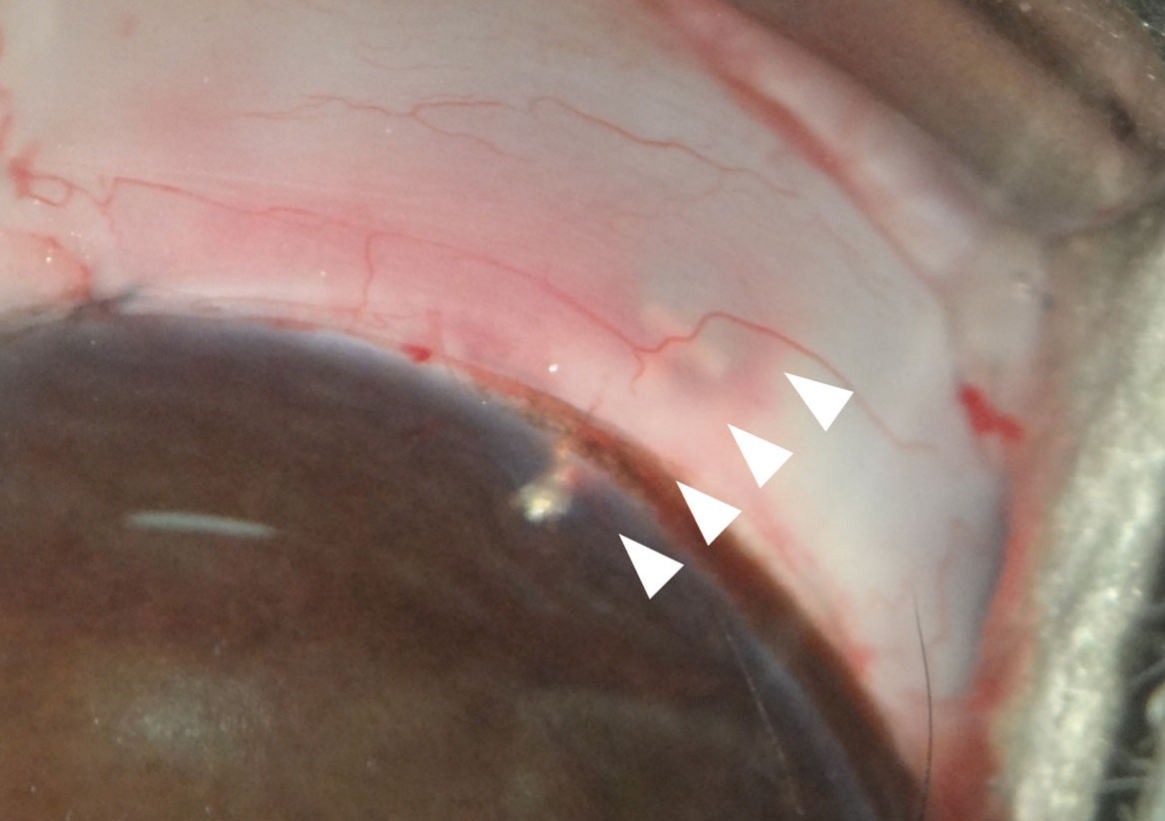
Anterior segment after Ex-PRESS shunt surgery. An Ex-PRESS shunt device (*arrowheads*) is inserted into the anterior chamber, and a bleb is well-formed under the conjunctiva.

**Figure 2.**
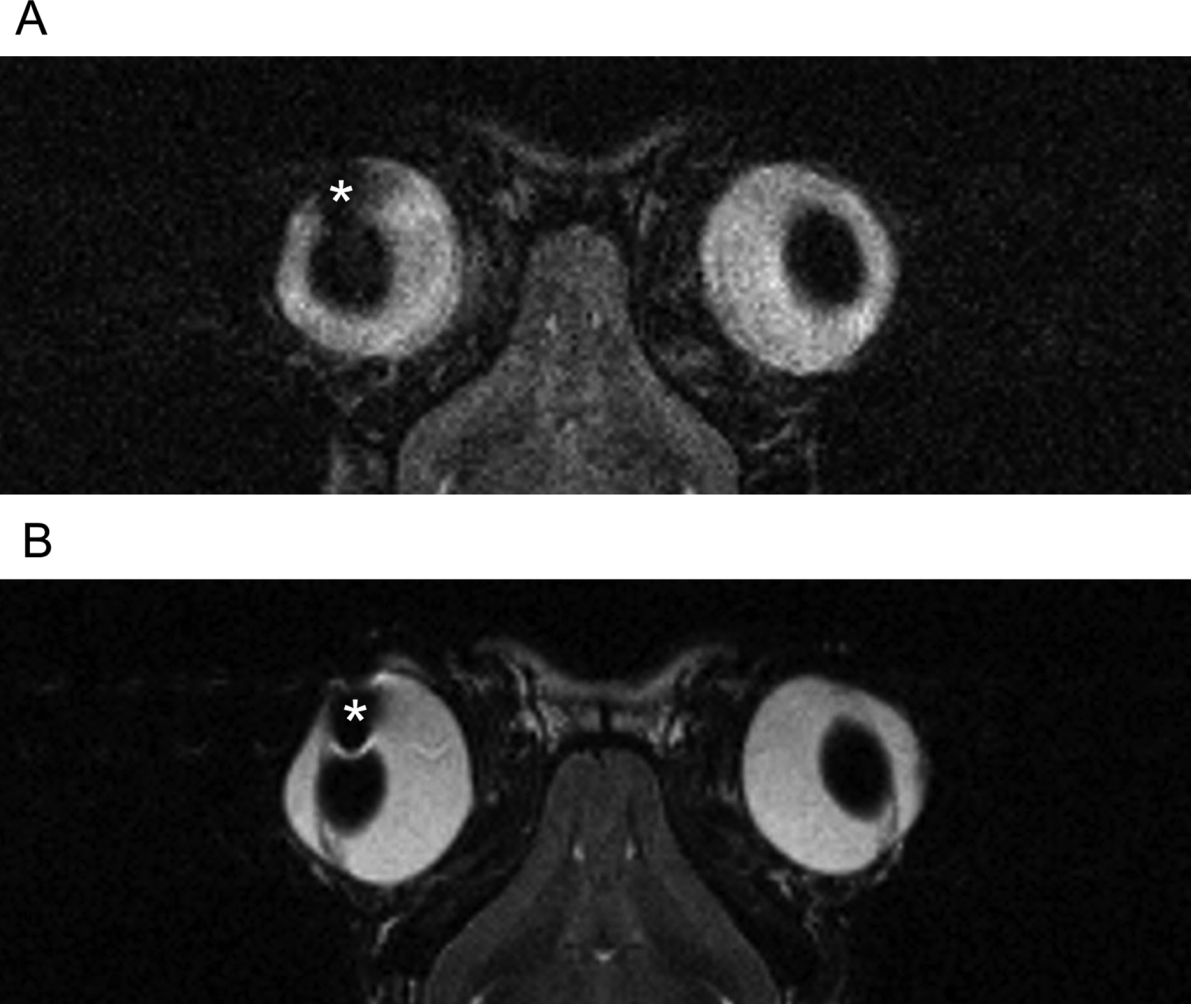
T2-weighted MR images of the Ex-PRESS-inserted eyes. **(A)** Image under low-SAR conditions. **(B)** Image under high-SAR conditions. * The Ex-PRESS insertion site. MR, magnetic resonance.

The Ex-PRESS glaucoma filtration device was inserted into one eye of each of the three rabbits in the MRI and non-MRI groups. A photograph of the anterior segment of the eye after Ex-PRESS insertion is shown in Figure 1. The Ex-PRESS was inserted into the anterior chamber, and a bleb was well-formed under the conjunctiva. Rabbits in the MRI group underwent a low-SAR MRI and a high-SAR MRI 1 and 2 weeks postoperatively, respectively. Images obtained with the high-SAR MRI were of a better quality than those obtained with a low-SAR MRI. There were no artifacts associated with the Ex-PRESS filtration device that interfered with image interpretation (Fig. 2). There was also no sign of dislocation of the Ex-PRESS device after MRI.

Changes in flare counts before and after Ex-PRESS shunt surgery are shown in Table 2. In the Ex-PRESS–inserted eyes, flare counts 2 hours after Ex-PRESS shunt surgery increased significantly compared with preoperative values (12.1 ± 3.8 pc/ms vs 241.3 ± 92.8 pc/ms, preoperation vs 2 hours after the operation, respectively; *P* < 0.01). The flare counts in the Ex-PRESS eyes decreased spontaneously to 85.9 ± 78.9 pc/ms and 22.4 ± 12.2 pc/ms on days 1 and 2 after surgery, respectively. In the non–Ex-PRESS-inserted eyes, the flare counts ranged from 10.3 ± 3.2 pc/ms to 12.2 ± 2.4 pc/ms, with no significant change between the preoperative and postoperative day 2 values.

**Table 2.** Summary of Aqueous Flare Counts and Ratios Before and After Ex-PRESS Shunt Surgery and MRI

	Aqueous Flare Counts (pc/ms)				Aqueous Flare Count Ratios (MRI/non-MRI Group)	
	MRI-Ex-PRESS	Non MRI-Ex-PRESS	MRI-Non Ex-PRESS	Non MRI-Non Ex-PRESS	Ex-PRESS	Non Ex-PRESS
Operation						
Before	12.3 ± 3.5	11.8 ± 4.8	10.1 ± 2.0	13.0 ± 4.8	1.3 ± 0.8	0.9 ± 0.3
2 hours	265.0 ± 71.2	217.6 ± 70.1	11.1 ± 3.1	13.3 ± 1.0	1.5 ± 0.6	0.8 ± 0.2
Day 1	97.3 ± 81.4	74.5 ± 92.4	9.7 ±2.9	11.0 ± 4.1	4.2 ± 3.2	1.9 ± 0.4
Day 2	25.9 ± 12.4	19.0± 13.5	11.0 ± 4.9	11.4 ± 4.5	2.1 ± 1.3	1.1 ± 0.5
Low-SAR MRI						
Before low-SAR MRI	11.3 ± 1.1	14.8 ± 4.8	13.1 ± 1.6	17.0 ± 6.6	0.8 ± 0.2	0.9 ± 0.4
2 hours	147.5 ± 108.0	14.3 ± 3.9	119.7 ± 81.3	13.9 ± 1.2	9.3 ± 5.0	8.4 ± 4.5
Day 1	11.33 ± 0.73	14.8 ± 4.6	10.0 ± 3.9	15.3 ± 3.2	0.8 ± 0.2	0.7 ± 0.2
Day 2	12.1 ± 3.4	14.6 ± 4.7	8.5 ± 0.8	11.6 ± 1.2	0.9 ± 0.2	0.7 ± 0.01
High-SAR MRI						
Before high-SAR MRI	12.0 ± 5.2	16.9 ± 6.5	14.6 ± 7.2	17.0 ± 4.9	0.8 ± 0.4	0.9 ± 0.4
2 hours	206.1 ± 83.8	76.8 ± 4.0	104.1 ± 48.9	77.4 ± 11.8	2.7 ± 0.8	1.4 ± 0.7
Day 1	16.9 ± 9.6	17.8 ± 5.1	8.9 ± 3.5	14.7 ± 7.2	0.9 ± 0.3	0.7 ± 0.2
Day 2	13.1 ± 3.0	15.3 ± 3.6	14.0 ± 3.4	13.3 ± 5.0	0.9 ± 0.1	1.1 ± 0.2

All values are presented as mean ± standard deviation. MRI, magnetic resonance imaging.

**Figure 3.**
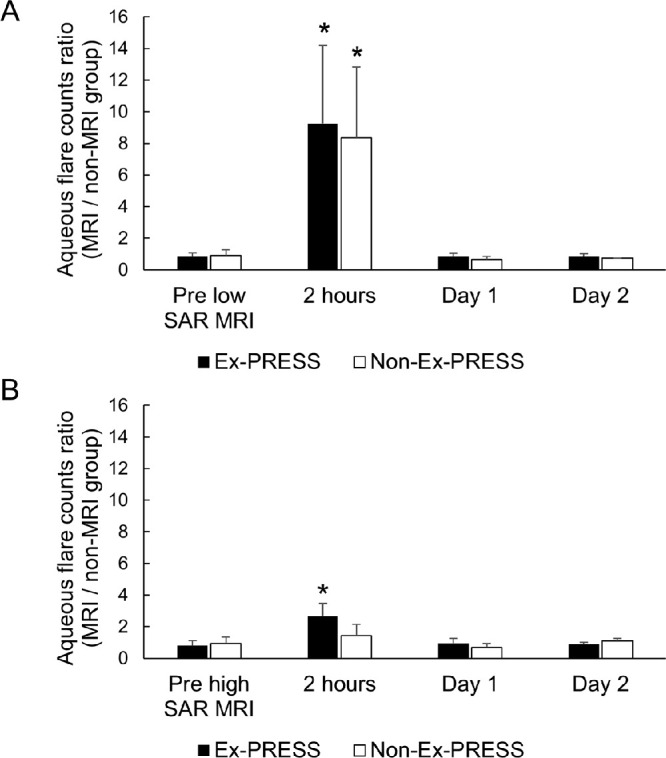
Changes in aqueous flare count ratios before and after MRI. **(A)** Changes in flare count ratios before and after MRI under low-SAR conditions. **(B)** Changes in flare count ratios before and after MRI under high-SAR conditions. Ex-PRESS, Ex-PRESS-inserted eyes; Non-Ex-PRESS, non–Ex-PRESS-inserted eyes. Mean ± SD are presented (*n* = 3). *P < 0.05. MRI, magnetic resonance imaging; SD, standard deviation.

Changes in flare counts and ratios before and after MRI are shown in Figure 3 and Table 2. The flare count ratio (MRI/non-MRI) 2 hours after MRI under low-SAR conditions in the Ex-PRESS eyes increased significantly compared with the pre–low-SAR MRI ratios (0.8 ± 0.2 vs 9.3 ± 4.9, pre–low-SAR MRI vs 2 hours after low-SAR MRI, respectively; *P* = 0.01). Flare count ratios decreased the day after low-SAR MRI and were not significantly different compared with pre–low-MRI ratios. The mean flare count ratio 2 hours after the low-SAR MRI in the non–Ex-PRESS-inserted eyes also increased compared with that of pre–low-SAR MRI (0.9 ± 0.4 vs 8.4 ± 4.5, pre–low-SAR MRI vs 2 hours after low-SAR MRI, respectively; *P* = 0.01). There was no significant difference in the flare count ratios between the Ex-PRESS and non–Ex-PRESS-inserted eyes at all time points (Fig. 3A).

The mean flare count ratio in the Ex-PRESS eyes 2 hours after MRI under high-SAR conditions increased significantly compared with that before high-SAR MRI (0.8 ± 0.3 vs 2.7 ± 0.8, pre–high-SAR MRI vs 2 hours after high-SAR MRI, respectively; *P* = 0.01). The flare count ratios decreased spontaneously the day after the high-SAR MRI. In contrast, there was no significant increase in flare count ratios after a high-SAR MRI in the non–Ex-PRESS-inserted eyes. Although the mean flare count ratio in the Ex-PRESS eyes 2 hours after a high-SAR MRI was approximately twice as high as that in the non–Ex-PRESS-inserted eyes, this difference was not statistically significant (2.7 ± 0.8 vs 1.4 ± 0.7, respectively; *P* = 0.18) (Fig. 3B). The mean flare count in the Ex-PRESS eyes 2 hours after high-SAR MRI was 206.1 ± 83.8 pc/ms. This value tended to be higher than 147.5 ± 108.0 pc/ms, which was the value 2 hours after low-SAR MRI in the same eyes; however, there was no significant difference (*P* = 0.50).

## Discussion

In this study, we showed that, in Ex-PRESS–inserted eyes, the aqueous flare counts in the anterior chamber 2 hours after MRI increased with high-field MRI. Flare counts decreased spontaneously the day after MRI, and there were no findings indicating Ex-PRESS dislocation after high-field MRI.

It has been shown that there was no deviation of the Ex-PRESS inserted in human cadaver eyes in a 3-T magnetic field.^15^ In this study, there was no obvious deviation of the Ex-PRESS. However, after low- and high-SAR MRI, there was an increase in anterior chamber flare in the Ex-PRESS–inserted eyes. The possible reasons for this finding may be the effects of invisible microvibrations occurring at a macroscopic level and device heat generation. A previous study reported that Ex-PRESS devices placed in a water-filled petri dish moved immediately across the dish in 1.5- and 3-T magnetic fields.^15^ Another study showed that Ex-PRESS devices placed on a petri dish showed extreme movement when exposed to a 4.7-T magnetic field.^17^ As these reports indicate, because the Ex-PRESS devices moved rapidly in the petri dishes, the Ex-PRESS devices inserted into the eyes in this study may have vibrated during 4.7-T MRI. It is assumed that microvibrations caused the implant device to stimulate the tissues near the iris and trabecular meshwork, causing an increase in flare counts. It is also commonly pointed out that MRI of patches that contain only small amounts of metal can cause burns.^21^^,^^22^ MRI has been reported to cause inflammation of surrounding tissues owing to heat generated by devices, such as deep brain stimulants and intracranial pressure monitoring devices.^23^^,^^24^ A previous study examined temperature changes in a 24-cm-long stainless steel nail used for intra-articular fixation. The nail was placed in a container filled with a gel with the same salinity as that of muscle tissue, and MRI was performed at a 4.0-W SAR with a 1.5-T MRI system. The results showed that the temperature increased more at the tip of the nail compared with that at the center of the nail.^25^ Because the Ex-PRESS has a cylindrical shape, its tip is susceptible to thermal stimulation. In particular, the anterior chamber of rabbit eyes is relatively shallow,^26^ and the heated tip of the Ex-PRESS can easily contact the iris, possibly causing inflammation.

In this study, the anterior chamber flare counts were increased 2 hours after low-SAR MRI in the non–Ex-PRESS-inserted eyes, although less than those in the Ex-PRESS–inserted eyes. If MRI itself might be unsafe for the eye, the flare counts should still have been increased 1 and 2 days after MRI. However, there was no increase 1 and 2 days after MRI, only after 2 hours. Although the cause of this observation is unclear, the interaction between general anesthesia with isoflurane and MRI may be a potential cause. A study of pediatric patients who underwent MRI showed that serum IL-1β, a marker of systemic inflammation, was significantly elevated after 60 minutes of anesthesia with isoflurane.^27^ Additionally, prolonged isoflurane anesthesia has also been reported to produce neuroinflammation.^28^

In this study, we investigated the safety of 4.7-T high-field MRI of Ex-PRESS–inserted eyes and noninserted eyes under two conditions: the low-SAR condition, which is SAR in accordance with the recommendations of international standards and safety guidelines, and the high-SAR condition, which is much higher than the guidelines recommendations. The results showed that intraocular inflammation was induced 2 hours after high-field MRI; however, this inflammation resolved spontaneously the next day without therapeutic intervention, such as with anti-inflammatory drugs. This temporary inflammation may have been influenced by inhalational anesthesia with isoflurane. However, inhalational anesthesia with isoflurane is not usually used in human MRI. This imaging is performed under awake conditions in adults who can remain at rest, and under oral or intravenous sedation in children and adults who cannot remain at rest. No deviation of the Ex-PRESS implants occurred during MRI imaging in either the low- or high-SAR conditions. This suggests that high-field MRI can be performed relatively safely in eyes implanted with Ex-PRESS shunt devices. MRI provides good visualization of soft tissues and is very useful for the early detection, diagnosis, and treatment of systemic diseases. Our study demonstrated the safety of high-field MRI in animal model after Ex-PRESS shunt surgery. This study suggests that high-field MRI imaging at 4.7 T could be relatively safe after Ex-PRESS shunt surgery.

## References

[1] Tham YC, Li X, Wong TY, Quigley HA, Aung T, Cheng CY. Global prevalence of glaucoma and projections of glaucoma burden through 2040: A systematic review and meta-analysis. *Ophthalmology*. 2014; 121(11): 2081–2090. 24974815 10.1016/j.ophtha.2014.05.013

[2] Heijl A, Leske MC, Bengtsson B, Hyman L, Bengtsson B, Hussein M. Reduction of intraocular pressure and glaucoma progression. *Arch Ophthalmol*. 2002; 120(10): 1268. 12365904 10.1001/archopht.120.10.1268

[3] Jonas JB, Aung T, Bourne RR, Bron AM, Ritch R, Panda-Jonas S. Glaucoma. *Lancet*. 2017; 390(10108): 2183–2193. 28577860 10.1016/S0140-6736(17)31469-1

[4] Kirwan JF, Lockwood AJ, Shah P, et al. Trabeculectomy in the 21st century: A multicenter analysis. *Ophthalmology*. 2013; 120(12): 2532–2539. 24070811 10.1016/j.ophtha.2013.07.049

[5] Scott IU, Greenfield DS, Schiffman J, et al. Outcomes of primary trabeculectomy with the use of adjunctive mitomycin. *Arch Ophthalmol*. 1998; 116(3): 286. 9514480 10.1001/archopht.116.3.286

[6] Bindlish R, Condon GP, Schlosser JD, D'Antonio J, Lauer KB, Lehrer R. Efficacy and safety of mitomycin-C in primary trabeculectomy: Five-year follow-up. *Ophthalmology*. 2002; 109(7): 1336–1341; discussion 1341–1332. 12093659 10.1016/s0161-6420(02)01069-2

[7] Sarkisian SR. The ex-press mini glaucoma shunt: Technique and experience. *Middle East Afr J Ophthalmol*. 2009; 16(3): 134–137. 20142979 10.4103/0974-9233.56226 PMC2813605

[8] Dahan E, Carmichael TR. Implantation of a miniature glaucoma device under a scleral flap. *J Glaucoma*. 2005; 14(2): 98–102. 15741808 10.1097/01.ijg.0000151688.34904.b7

[9] Maris PJ Jr., Ishida K, Netland PA. Comparison of trabeculectomy with Ex-PRESS miniature glaucoma device implanted under scleral flap. *J Glaucoma*. 2007; 16(1): 14–19. 17224744 10.1097/01.ijg.0000243479.90403.cd

[10] Ishida K, Moroto N, Murata K, Yamamoto T. Effect of glaucoma implant surgery on intraocular pressure reduction, flare count, anterior chamber depth, and corneal endothelium in primary open-angle glaucoma. *Jpn J Ophthalmol*. 2017; 61(4): 334–346. 28374269 10.1007/s10384-017-0512-2

[11] Chen G, Li W, Jiang F, Mao S, Tong Y. Ex-PRESS implantation versus trabeculectomy in open-angle glaucoma: A meta-analysis of randomized controlled clinical trials. *PLoS One*. 2014; 9(1): e86045. 24465860 10.1371/journal.pone.0086045 PMC3900454

[12] Wang W, Zhang X. Meta-analysis of randomized controlled trials comparing EX-PRESS implantation with trabeculectomy for open-angle glaucoma. *PLoS One*. 2014; 9(6): e100578. 24972022 10.1371/journal.pone.0100578 PMC4074054

[13] Mabray MC, Uzelac A, Talbott JF, Lin SC, Gean AD. Ex-PRESS glaucoma filter: An MRI compatible metallic orbital foreign body imaged at 1.5 and 3T. *Clin Radiol*. 2015; 70(5): e28–e34. 25735675 10.1016/j.crad.2015.01.010 PMC4385419

[14] Medicines and Healthcare products Regulatory Agency. Guidelines for magnetic resonance equipment in clinical use. 2021. Available at: https://www.gov.uk/government/publications/safety-guidelines-for-magnetic-resonance-imaging-equipment-in-clinical-use.

[15] Geffen N, Trope GE, Alasbali T, Salonen D, Crowley AP, Buys YM. Is the Ex-PRESS glaucoma shunt magnetic resonance imaging safe? *J Glaucoma*. 2010; 19(2): 116–118. 19661826 10.1097/IJG.0b013e3181a98bda

[16] De Feo F, Roccatagliata L, Bonzano L, Castelletti L, Mancardi G, Traverso CE. Magnetic resonance imaging in patients implanted with Ex-PRESS stainless steel glaucoma drainage microdevice. *Am J Ophthalmol*. 2009; 147(5): 907–911.e901. 19232564 10.1016/j.ajo.2008.12.011

[17] Seibold LK, Rorrer RAL, Kahook MY. MRI of the Ex-PRESS stainless steel glaucoma drainage device. *Br J Ophthalmol*. 2011; 95(2): 251–254. 20576770 10.1136/bjo.2009.173906

[18] De Vita E, Thomas DL, Roberts S, et al. High resolution MRI of the brain at 4.7 Tesla using fast spin echo imaging. *Br J Radiol*. 2003; 76(909): 631–637. 14500278 10.1259/bjr/69317841

[19] Bromley LRA, De Saussure VA, Clipp JC, Wright JS. Heat capacities of sea water solutions at salinities of 1 to 12% and temperatures of 2.degree. to 80.degree. *J Chem Eng Data*. 1967; 12(2): 202–206.

[20] Shellock FG. Radiofrequency energy-induced heating during MR procedures: A review. *J Magn Reson Imaging*. 2000; 12(1): 30–36. 10931562 10.1002/1522-2586(200007)12:1<30::aid-jmri4>3.0.co;2-s

[21] Karch AM. Don't get burnt by the MRI: Transdermal patches can be a hazard to patients. *Am J Nurs*. 2004; 104(8): 31. 10.1097/00000446-200408000-00023 15300037

[22] Paparella S. Transdermal patches: An unseen risk for harm. *J Emerg Nurs*. 2005; 31(3): 278–281. 15983583 10.1016/j.jen.2005.01.010

[23] Henderson JM, Tkach J, Phillips M, Baker K, Shellock FG, Rezai AR. Permanent neurological deficit related to magnetic resonance imaging in a patient with implanted deep brain stimulation electrodes for Parkinson's disease: Case report. *Neurosurgery*. 2005; 57(5): E1063. 16284543 10.1227/01.neu.0000180810.16964.3e

[24] Tanaka R, Yumoto T, Shiba N, et al. Overheated and melted intracranial pressure transducer as cause of thermal brain injury during magnetic resonance imaging: Case report. *J Neurosurg*. 2012; 117(6): 1100–1109. 23061386 10.3171/2012.9.JNS12738

[25] Muranaka H, Horiguchi T, Ueda Y, Tanki N. Evaluation of RF heating due to various implants during MR procedures. *Magn Reson Med Sci*. 2011; 10(1): 11–19. 21441723 10.2463/mrms.10.11

[26] Sorsby A, Stone J, Leary GA, Sheridan M. Changes in the depth of the anterior chamber and in the radius of curvature of the front surface of the lens during growth observations on the rabbit *Br J Ophthalmol*. 1960; 44(8): 467–471. 13832934 10.1136/bjo.44.8.467 PMC509971

[27] Whitaker EE, Christofi FL, Quinn KM, et al. Selective induction of IL-1β after a brief isoflurane anesthetic in children undergoing MRI examination. *J Anesth*. 2017; 31(2): 219–224. 28050702 10.1007/s00540-016-2294-y

[28] Peng L, Liu S, Xu J, et al. Metformin alleviates prolonged isoflurane inhalation induced cognitive decline via reducing neuroinflammation in adult mice. *Int Immunopharmacol*. 2022; 109: 108903. 35709590 10.1016/j.intimp.2022.108903 PMC9190296

